# The Effect of Oxygen Vacancies on the Diffusion Characteristics of Zn(II) Ions in the Perovskite SrTiO_3_ Layer: A Computational Study

**DOI:** 10.3390/ma16113957

**Published:** 2023-05-25

**Authors:** Yong Nam Ahn

**Affiliations:** Department of Chemical and Biological Engineering, Gachon University, Seongnam 13120, Gyeonggi, Republic of Korea; yahn@gachon.ac.kr

**Keywords:** strontium titanate, Zn(II) ion diffusion, oxygen vacancies, anode coating, aqueous zinc-ion batteries

## Abstract

A highly polar perovskite SrTiO_3_ (STO) layer is considered as one of the promising artificial protective layers for the Zn metal anode of aqueous zinc-ion batteries (AZIBs). Although it has been reported that oxygen vacancies tend to promote Zn(II) ion migration in the STO layer and thereby effectively suppress Zn dendrite growth, there is still a lack of a basic understanding of the quantitative effects of oxygen vacancies on the diffusion characteristics of Zn(II) ions. In this regard, we comprehensively studied the structural features of charge imbalances caused by oxygen vacancies and how these charge imbalances affect the diffusion dynamics of Zn(II) ions by utilizing density functional theory and molecular dynamics simulations. It was found that the charge imbalances are typically localized close to vacancy sites and those Ti atoms that are closest to them, whereas differential charge densities close to Sr atoms are essentially non-existent. We also demonstrated that there is virtually no difference in structural stability between the different locations of oxygen vacancies by analyzing the electronic total energies of STO crystals with the different vacancy locations. As a result, although the structural aspects of charge distribution strongly rely on the relative vacancy locations within the STO crystal, Zn(II) diffusion characteristics stay almost consistent with changing vacancy locations. No preference for vacancy locations causes isotropic Zn(II) ion transport inside the STO layer, which subsequently inhibits the formation of Zn dendrites. Due to the promoted dynamics of Zn(II) ions induced by charge imbalance near the oxygen vacancies, the Zn(II) ion diffusivity in the STO layer monotonously increases with the increasing vacancy concentration ranging from 0% to 16%. However, the growth rate of Zn(II) ion diffusivity tends to slow down at relatively high vacancy concentrations as the imbalance points become saturated across the entire STO domain. The atomic-level understanding of the characteristics of Zn(II) ion diffusion demonstrated in this study is expected to contribute to developing new long-life anode systems for AZIBs.

## 1. Introduction

Due to their high energy density, extended lifespan, and dependable performance, lithium-ion batteries (LIBs) have dominated the secondary energy storage industry over the past few decades. However, there are significant concerns, especially for large-scale energy storage systems, arising from the high flammability and toxicity of organic electrolytes, insufficient Li sources, and cell production complexity [1,2,3,4,5,6]. Due to the relatively higher abundance of sodium and potassium and their similar chemical characteristics to those of lithium, sodium-ion batteries (SIBs) and potassium-ion batteries (KIBs) have also been the subject of extensive research [7,8,9,10,11]. Nevertheless, SIBs and KIBs utilize extremely toxic electrolytes, similar to LIBs, raising safety issues and driving up the cost of fabrication. 

Aqueous rechargeable batteries have a significantly higher competitive potential for energy storage devices compared to batteries that use organic electrolytes because of their exceptional benefits, including (i) high safety due to the absence of hazardous solvents with high volatility, (ii) low cost and environmental friendliness, and (iii) high ionic conductivity of aqueous electrolytes compared to that of organic electrolytes [12,13,14,15,16,17]. As a result, extensive research efforts have been made to explore the various types of aqueous rechargeable batteries including zinc-ion batteries (AZIBs) [18,19,20,21,22], magnesium-ion batteries (AMIBs) [23], and aluminum-ion batteries (AAIBs) [24]. In particular, AZIBs have developed into competitive alternatives to LIBs due to their low redox potential (−0.762 V vs. a standard hydrogen electrode), high theoretical capacity (819 mAh/g and 5849 mAh/cm^3^), abundant natural resources, low cost, and ease of production [25,26,27]. Despite these beneficial aspects, AZIBs still suffer from various drawbacks such as uncontrollable dendrite growth, parasitic corrosion reactions, and water-related side reactions [28,29]. One of the primary barriers to the widespread commercialization of AZIBs in particular is “Zn dendrites” which are associated with non-uniform deposition of Zn(II) ions on Zn metal, which cause short-circuiting by puncturing the separator [30].

Since Zn dendrites grow at electrode–electrolyte interfaces, forming an artificial protective layer on Zn metal with materials such as metals [31,32], inorganic semiconductors [33,34,35,36,37,38], and insulating polymers [14,39] has garnered considerable interest as one of the most promising methods to stop dendrite growth by physically obstructing the direct contact between the electrolyte and Zn metal. In particular, Han et al. decorated a Zn surface with a metallic indium (In) layer by dipping Zn foils into an aqueous InCl_3_ solution [31]. According to the report, In serves as a nucleating agent for the uniform deposition of Zn, which inhibits the development of Zn dendrites. Zeng et al. suggested a flexible 3D carbon nanotube (CNT) framework for Zn/CNT anodes in order to prevent the growth of Zn dendrites through a uniformly distributed electric field brought on by the high electric conductivity of the CNT [40]. Recently, Ko et al. utilized a highly polar perovskite SrTiO_3_ (STO) as the protective layer on Zn metal [41]. Along with the uniform Zn(II) ion rearrangement brought on by STO’s high dielectric constant, the authors noted that the promoted Zn(II) ion diffusivity brought on by charge imbalance near oxygen vacancies in the STO layer is another favorable factor to reduce dendrite growth and improve the durability of AZIBs. The study demonstrated that oxygen vacancies increase Zn(II) ion kinetics in the STO layer, leading to the excellent electrochemical performance of AZIBs. In particular, CV measurements employing a symmetric cell at a scan rate of 1.0 mV/s reveal that the gradient of a Zn anode with an STO coating layer is roughly two orders of magnitude larger than that of a bare Zn anode (36.42 mA/V vs. 0.33 mA/V). This increased gradient of the STO-coated Zn anode system is a direct result of accelerated Zn(II) ion diffusion, resulting in enhanced Zn ion electrochemical kinetics. Therefore, it is anticipated that by using controlled oxygen vacancies in the protective coating layers, a promising anode system for advanced Zn-metal batteries can be developed. To this end, it is essential to have a thorough knowledge of how oxygen vacancies affect the way that Zn(II) ions diffuse in the STO layer.

Since oxygen vacancies play a crucial role in numerous features of perovskite materials, the effects of oxygen vacancies on various physical properties such as atomic structure, electrical properties, and diffusion characteristics have been extensively investigated [42,43,44,45]. Through the use of atomic-resolution transmission electron microscopy and density functional calculations, Jin et al. investigated the impact of oxygen vacancies on the atomic configuration of perovskite cobaltite films [42]. They reported that AO–AO (A = Pr/Ba) interplanar spacings are linearly correlated with the oxygen vacancy concentration in the enclosing Co–O planes. When LaAlO_3_ (LAO) films were deposited on STO substrates, Kalabukhov et al. found that the oxygen vacancies created in the STO substrates as the LAO films grow induce high electrical conductivity and mobility values at the LAO/STO interfaces [43]. Regarding the diffusion phenomena in perovskite oxides, De Souza comprehensively summarized the theoretical background of various forms of ion transport as well as experimental approaches to obtain diffusion coefficients [44]. In addition, Zhong et al. reported that the migration of oxygen vacancies in STO leads to a current–voltage characteristics curve such as the resistive switching effect at high temperatures [45]. However, despite this noticeable progress, to the best of the author’s knowledge, there has been no research focusing on the effect of oxygen vacancies in perovskite materials on Zn(II) ion diffusion.

In this regard, we systematically investigated how the oxygen vacancies in the STO layer affect the characteristics of Zn(II) ion dynamics at the atomic level. Based on an earlier study that found that the charge imbalance caused by the oxygen vacancies encourages Zn(II) ion diffusivity [41], the precise structure of the electron density close to the oxygen vacancies was examined by utilizing density functional theory (DFT) calculations. The spatial distribution of the charge imbalance strongly relies on the relative locations of the oxygen vacancies within the STO crystal because the charge imbalance tends to be localized near the vacancies. As a result, we investigated the structural stability of STO systems with various vacancy locations and how these relative stabilities between the various vacancy locations impact the Zn(II) ion diffusivity in the STO layer. The concentration of oxygen vacancies, in addition to the vacancy locations, is another crucial element that modifies the charge imbalance morphology within the STO layers. Therefore, using molecular dynamics (MD) simulations, the impact of the vacancy concentration on Zn(II) ion diffusivity was quantitatively analyzed. This multiscale computational approach allows us to understand the comprehensive influence of the oxygen vacancies on Zn(II) ion dynamics in the STO layer and can thus bring advances in the development of new anode systems for AZIBs with long lifetimes.

## 2. Computational Details

First-principles calculations were utilized to find the electronic total energies and charge densities (Figure S1) of the considered perovskite structures in this study. Vienna ab-initio Simulation Package (VASP) [46,47] was used for the calculations, and the Perdew–Burke–Ernzerhof (PBE) function of the generalized gradient approximation (GGA) was adopted for the exchange and correlation contributions [48]. Core electrons were incorporated into the pseudopotentials using the projector augmented wave (PAW) method [49]. A 2 × 2 × 1 supercell containing 20 atoms/cell was generated, and the 16% oxygen vacancies observed in the previous study [41] were introduced into the structure by removing two oxygen atoms. A Plane-wave cutoff energy of 520 eV was used with a 4 × 4 × 8 k mesh in terms of the Monhorst–Pack scheme [50] for Brillouin zone integration. Structural optimization was stopped until the energy convergence and atomic force were lower than 10^−5^ eV and 0.02 eV/Å, respectively. 

The diffusion characteristics of Zn(II) ions in the considered perovskite structures were investigated by utilizing MD simulations. From the atomic trajectories of the MD simulations, the mean squared displacement (MSD) of a Zn(II) ion can be calculated as:

(1) $$\mathrm{MSD}={\langle \left(r\left(t\right)-r_{0}\right)\rangle }^{2}$$

where $r\left(t\right)$ is the position of the Zn(II) ion at time t, and $r_{0}$ is its initial position. The diffusion coefficient (D) of the Zn(II) ion can be evaluated from the slope of the linear regression of the time–MSD curve as follows:

(2) $$D=\frac{\mathrm{MSD}}{6t}$$

The initial MD configuration of the system for Zn(II) ion diffusion was prepared by putting a Zn atom into an arbitrary interstitial site of a perovskite structure whose size is 8 × 8 × 4 nm^3^. During all MD simulations, the temperature was kept constant at 1400 K using a Nose–Hoover thermostat [51,52] to effectively capture the dynamic characteristics of the Zn(II) ion within the MD time scale. The pressure of the systems was fixed at 1 atm via a Parrinello–Rahman barostat [53,54]. All MD simulations in this study were performed by using Large-Scale Atomic/Molecular Massively Parallel Simulator (LAMMPS) [55,56] with interatomic potential developed by Pedone et al. (Table S1) [57]. Individual MD simulations were performed for 10 ns with a 1 fs integration time step. After initial equilibrations for 1 ns, the MSDs of the Zn(II) ion were estimated as a function of time for a 100 ps time length. The diffusion coefficients were predicted by performing a linear regression of the time–MSD curve from 10 to 100 ps. 

## 3. Results and Discussion

### 3.1. Effect of Charge Imbalance Structure on Zn(II) Ion Diffusion

**Figure 1:**
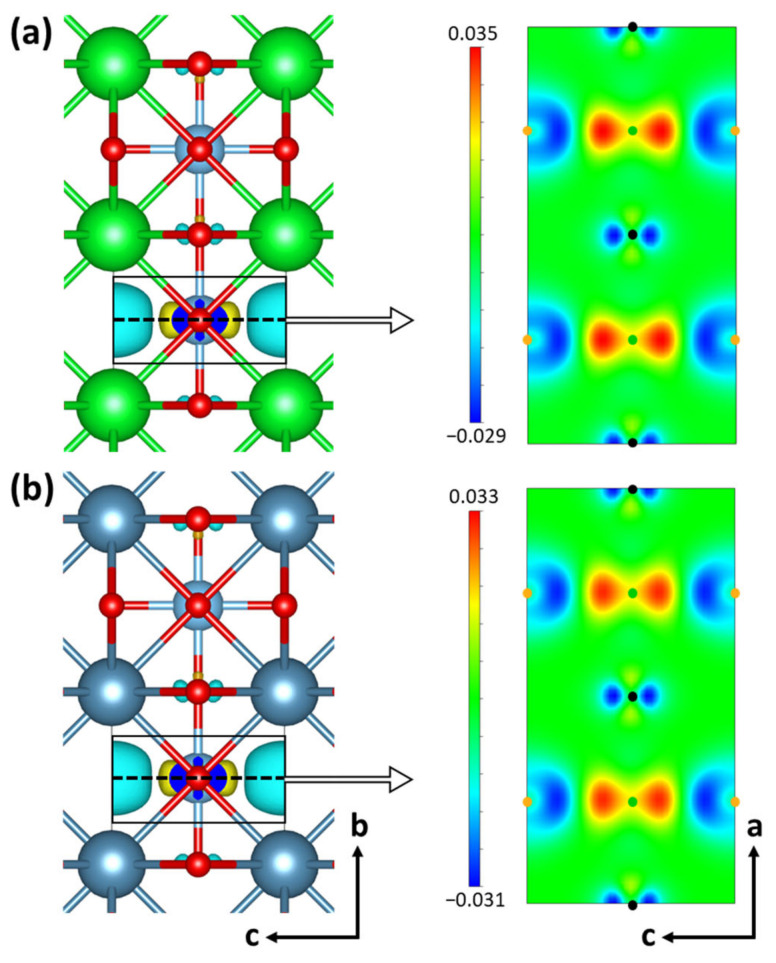
Differential electron density distribution of (**a**) SrTiO_3_ and (**b**) CaTiO_3_. The Sr, Ca, Ti, and O atoms are denoted by green, dark blue, light blue, and red, respectively. The yellow cloud represents electron accumulation after introducing an oxygen vacancy, and the cyan cloud indicates a negative electron isosurface. For each system, a 2D map is presented for the cross-section located at the oxygen vacancies. On the 2D maps, the locations of Ti, O, and O vacancies are denoted by green, black, and orange dots, respectively.

Zn(II) ions diffuse through the STO layer after being absorbed by the electrolyte on the outer STO surface, eventually depositing on the Zn anode surface. It was shown in an earlier study [41] that oxygen vacancies in the STO layer lead to unequal charge distribution, which subsequently increases Zn(II) ion diffusivity. Figure 1a presents differential electron density distribution caused by oxygen vacancies in the STO layer obtained from DFT calculations. The electron density around the introduced oxygen vacancies reduces as a result of the absence of oxygen, which has a high electronegativity, and electrons end up accumulating at the nearby Ti atoms as a result. It is noteworthy that this change in electron density brought on by the oxygen vacancies happens rather locally, i.e., the changes in electron density are restricted to the oxygen vacancies and the nearby Ti atoms, and the electron structure around all other atoms in the system essentially stays the same. This shows that of the three elements in the STO system, Ti and O play critical roles in charge imbalance caused by oxygen vacancies, whereas Sr has an almost negligible impact.

By examining the differential electron density distribution brought on by oxygen vacancies in the CaTiO_3_ (CTO) system, the observed differentiable effects of each element in STO on the charge imbalance are further validated. The variations in electron density brought on by oxygen vacancies in CTO are shown in Figure 1b, and they are also restricted to the vicinity of the vacancies and the nearby Ti atoms. Along with the domain in which the oxygen vacancy effectively alters the electron density, the STO and CTO systems exhibit nearly identical electron density changes in terms of magnitude as well. The maximum and minimum values for the variations in electron density in the STO and CTO systems are listed in Table 1. Less than 7% separates the maximum values of the two systems. The minimum value differences between the two systems are similarly smaller than 7%. Furthermore, the gap between the maximum and minimum (represented by ΔQ in Table 1), which measures the extent of charge imbalance, is the same in both systems.

**Figure 2:**
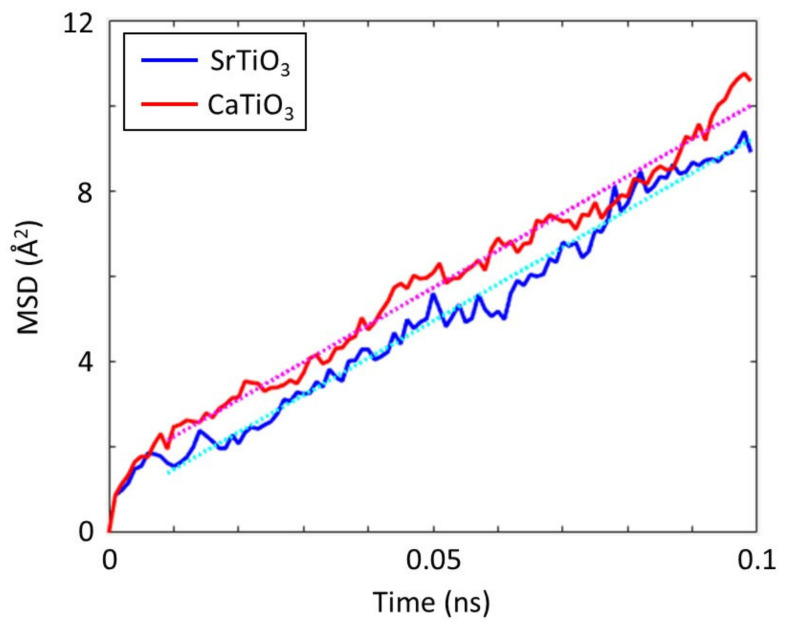
Mean squared displacements (MSDs) of Zn(II) ion in SrTiO_3_ and CaTiO_3_ with 16% oxygen vacancies. The dotted lines present linear regressions of MSDs from 0.01 ns to 0.1 ns.

Because both STO and CTO have the same charge imbalance, it follows that the impact of oxygen vacancies on Zn(II) ion diffusivity will be nearly equivalent in both systems. By using MD simulations, the MSD of Zn(II) ions in the two systems with the same oxygen concentration is determined in order to compare the diffusivity of Zn(II) ions in the STO and CTO systems. The concentration of oxygen vacancies for the simulations is set at 16% in accordance with the experimental findings in a previous study [41]. As seen in Figure 2, the resulting MSDs do not clearly distinguish between the two systems, which leads to essentially identical Zn(II) ion diffusion coefficients (14.45 Å^2^/ns and 14.52 Å^2^/ns in STO and CTO, respectively). This finding further demonstrates that the charge imbalance brought on by oxygen vacancies is a major determinant of the Zn(II) ion transport properties.

### 3.2. Effect of Vacancy Locations on Zn(II) Ion Diffusion

**Figure 3:**
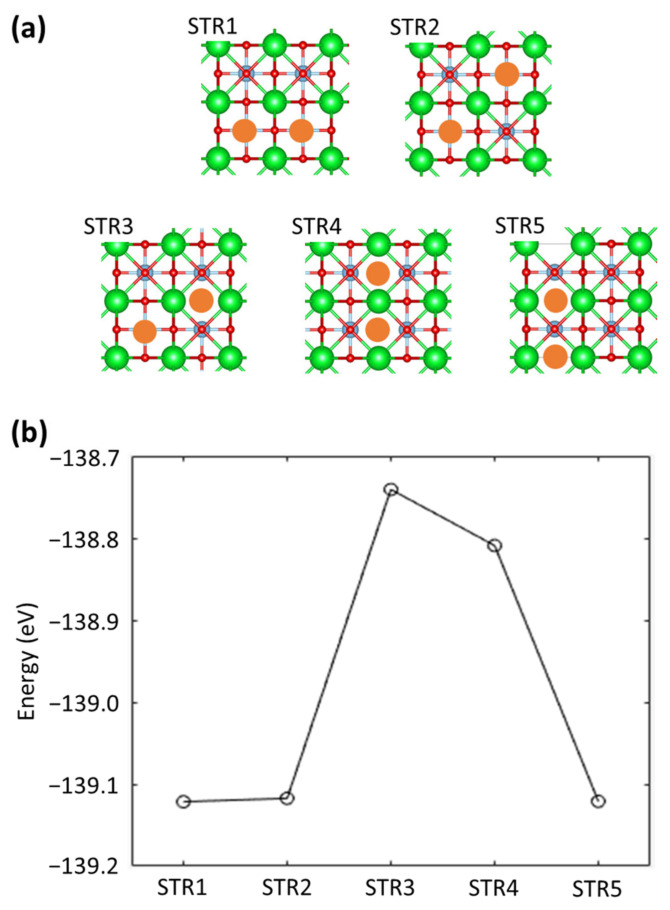
(**a**) Five atomic configurations with two oxygen vacancies in SrTiO_3_. The Sr, Ti, and O atoms are denoted by green, light blue, and red, respectively. The orange circles present the locations of the oxygen vacancies. (**b**) Corresponding electronic total energies to the five atomic configurations.

Since Section 3.1 showed that differential electron density is highly localized at oxygen vacancies, it is anticipated that the relative positions of the oxygen vacancies inserted into the crystal will have an impact on the direction and magnitude of Zn(II) ion diffusion in the STO system. By removing two oxygen atoms from the 2 × 2 × 1 supercell, an STO system with 16% oxygen vacancies can be created. Five differentiable atomic configurations with two oxygen vacancies were built by taking structural symmetry into account, as illustrated in Figure 3a. Then, the electronic total energies of the five systems were evaluated in order to examine the relative stability of the constructed structures. The calculated energies, which are displayed in Figure 3b, show that there are only minor energy differences between the structures. Specifically, the energy difference between STR1 (the most stable structure) and STR3 (the least stable structure) is 0.021 eV/atom, which can be overcome by thermal energy at room temperature.

**Figure 4:**
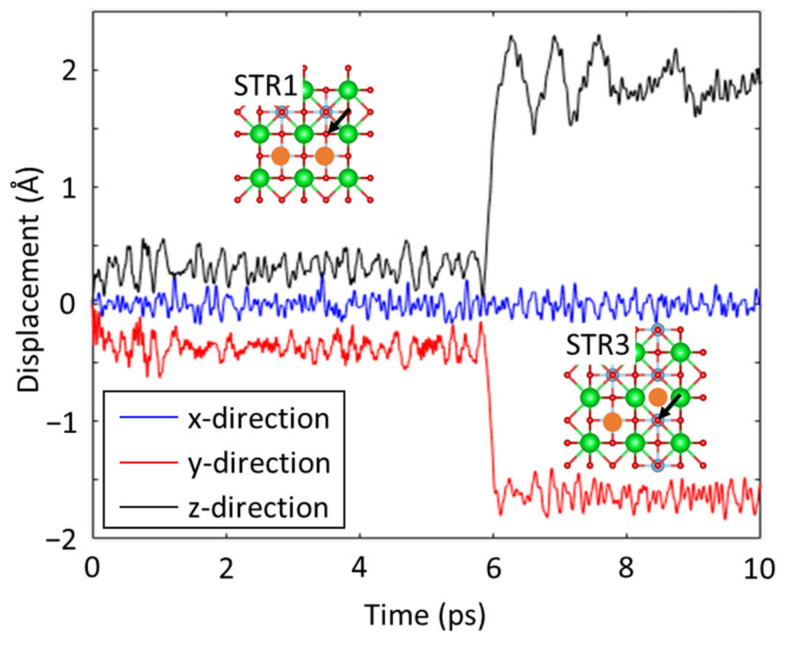
Displacement of the oxygen atom marked with arrows in the insets during an equilibrium MD simulation at 300 K temperature.

The minimal energy reliance on the position of the vacancy is further confirmed by performing a straightforward MD simulation. The simulation starts with the structure denoted by STR1, which has the lowest energy among the five structures shown in Figure 3. The simulation is run at a constant temperature of 300 K while tracing the coordination of a particular oxygen atom indicated by an arrow in the insets of Figure 4. During the simulation, the specified atom’s displacement from its initial position in the x-, y-, and z-directions as a function of time is presented in Figure 4. After initial vibration for approximately 6 ps, the oxygen atom hopped to one of the vacancy locations. Since this oxygen hopping consequently transformed the structure with the lowest energy (i.e., STR1) into the structure with the highest energy (i.e., STR3), it was further confirmed that there is no obvious locational preference for oxygen vacancies in the STO system.

**Figure 5:**
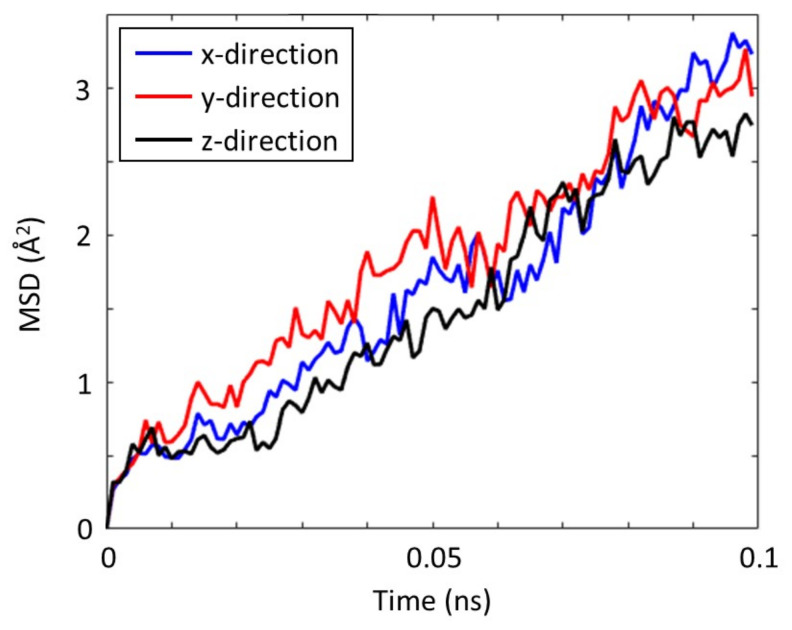
Mean squared displacement (MSD) of Zn(II) ions in SrTiO_3_ with 16% oxygen vacancies. The MSDs are separately calculated for the x-, y-, and z-directions.

Since the oxygen vacancies in STO have no preference for location, this indicates that the Zn(II) ion diffusion caused by the charge imbalance from the oxygen vacancies has no preference for direction too. To determine whether Zn(II) ions have a predilection for diffusion in any particular direction, the MSDs of Zn(II) ions in the x-, y-, and z-directions were individually calculated. The measured MSDs in each direction are nearly comparable to one another as seen in Figure 5. Therefore, regardless of where the oxygen vacancies are in the STO crystal, they cause an isotropic augmentation of Zn(II) ion diffusivity.

### 3.3. Effect of Vacancy Concentration on Zn(II) Ion Diffusion

Since oxygen vacancies effectively promote Zn(II) ion diffusivity in the STO system, it is anticipated that Zn(II) ion diffusivity in STO can be successfully regulated by modifying the concentration of oxygen vacancies. In order to achieve this, it is critical to look at the quantitative relationship between Zn(II) ion diffusivity and oxygen vacancy concentration. Five STO structures with vacancy concentrations ranging from 0% to 16% were built in order to investigate diffusion characteristics of the Zn(II) ion at various oxygen vacancy concentrations. The slopes of MSDs computed during MD simulations were then used to estimate the Zn(II) ion diffusion coefficients in the five prepared STO structures.

**Figure 6:**
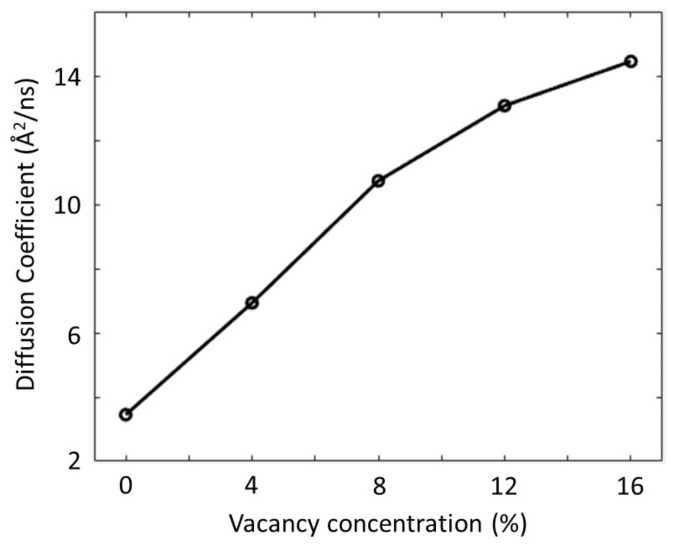
Diffusion coefficient of Zn(II) ions in SrTiO_3_ as a function of oxygen concentrations ranging from 0% to 16%.

The calculated diffusion coefficients monotonically grow with increasing oxygen vacancies in STO, as seen in Figure 6. When the concentration of oxygen vacancies is relatively low, the number of charge imbalance sites would be practically proportional to the concentration because the charge imbalance caused by oxygen vacancies tends to cluster near the vacancy locations. The influence of oxygen vacancies upon charge imbalance, however, eventually diminishes at high vacancy concentrations as the imbalance locations inside STO become saturated as the concentration rises. As a result, as the vacancy content rises, the rate of rising Zn(II) ion diffusivity tends to decrease, as seen in Figure 6.

**Figure 7:**
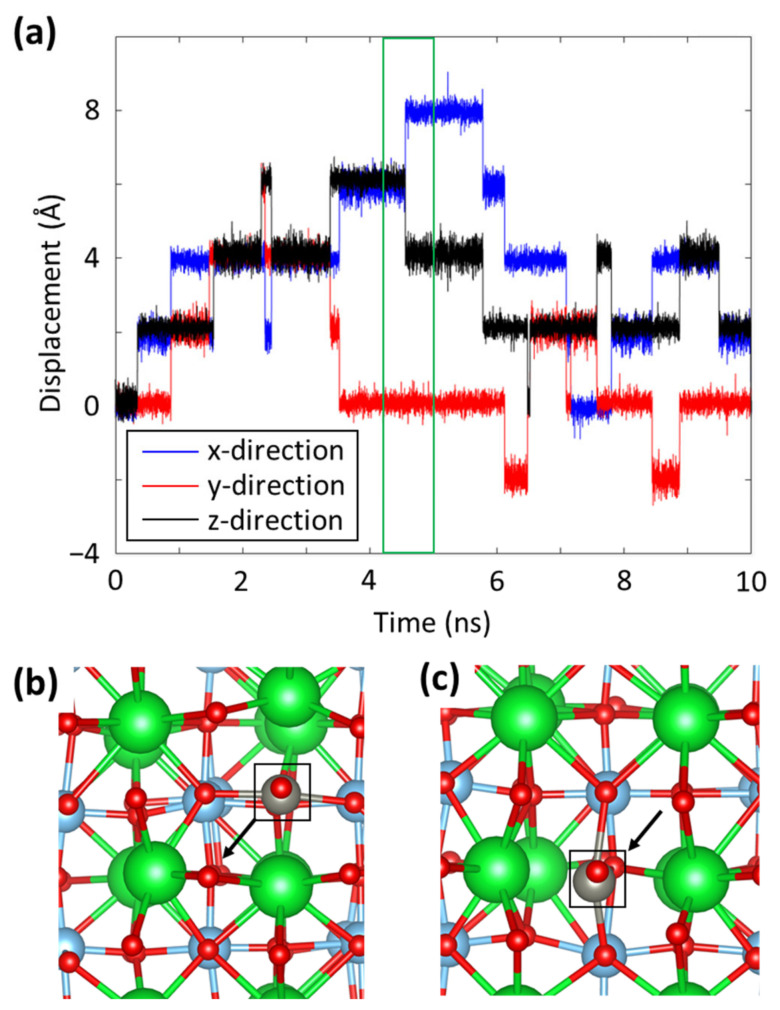
(**a**) Displacement of a Zn(II) ion in SrTiO_3_ during an equilibrium MD simulation, and atomic configurations of the system (**b**) before and (**c**) after a position jump of the Zn(II) ion that happens at 4.6 ns. Sr, Ti, O, and Zn are denoted by green, light blue, red, and grey, respectively. The arrow points in the direction of the jump, and squares represent the locations of the Zn(II) ions.

We note that the Zn(II) ion has a non-zero value of diffusion coefficient (3.45 Å^2^/ns) even at a 0% vacancy concentration in the STO layer. This can be explained by tracking the Zn(II) ion coordination. Figure 7a shows the Zn(II) ion displacement from its initial position in the x-, y-, and z-directions as a function of time during the performed MD simulation for the STO system with 0% oxygen vacancy. As shown, the introduced Zn(II) ion spends most of the simulation time with vibrational motion around several specific locations. These locations are meta-stable interstitial sites for the Zn(II) ion to stay in the STO structure. Intermittently, thermal energy that can overcome the energy barrier of a meta-stable site is transferred to the Zn(II) ion from the adjacent atoms during the vibrational motion; then, the Zn(II) ion jumps to one of the neighboring meta-stable sites. Atomic configurations before and after this Zn(II) ion jump between two neighboring interstitials are presented in Figure 7b,c, respectively. As a result of the integration of these stochastic jumps, the Zn(II) ion has a non-zero diffusion coefficient value. Since charge imbalance caused by oxygen vacancies can provide additional deriving force to the described Zn(II) ion jumps, the diffusion coefficient of the Zn(II) ion increases with the increasing vacancy concentration in STO.

## 4. Conclusions

In this study, we systematically investigated the structural aspects of the charge imbalance brought on by oxygen vacancies in STO systems, as well as how these aspects affect the diffusivity of Zn(II) ions at the atomic level. It has been found that changes in electron density brought on by oxygen vacancies only affect the nearby Ti atoms and the vacancies themselves, with changes occurring nearly nowhere else. According to this discovery, charge imbalances created by oxygen vacancies are confined, and as a result, the augmentation of Zn(II) ion diffusivity caused by this charge imbalance only manifests itself in the vicinity of the vacancies. Through examination of differential electron density and Zn(II) ion diffusivity in CTO systems, this localized charge imbalance was further validated. The Zn(II) ion diffusion affected by the charge imbalances exhibits essentially isotropic mobility in all directions regardless of the vacancy sites, despite the charge imbalances clustered around oxygen vacancies suggesting that their distribution features depend substantially on the vacancy locations. The isotropic diffusion of Zn(II) ions occurs in the STO system as a result of dynamic changes in vacancy locations because the energy difference between various vacancy positions can be easily overcome by thermal energy, even at room temperature. Due to the localization of charge imbalance around oxygen vacancies, the number of imbalance spots is proportional to the vacancy concentration. As a result, the Zn(II) ion diffusivity elevates with increasing vacancy concentration. However, as the imbalance spots become saturated throughout the entire STO domain, the growth rate tends to slow down at relatively large vacancy concentrations.

Effective control of Zn(II) ion diffusion by coating materials on an anode surface is crucial to prevent the formation of Zn dendrites. The key findings regarding the impact of oxygen vacancies on Zn(II) ion diffusivity in the STO coating layer presented in this study can offer some suggestions for the fabrication of novel coating materials on anode surfaces for developing advanced AZIBs. Specifically:

(i) The effects of oxygen vacancies on Zn(II) ion diffusivity may stay the same in other perovskite materials such as CaTiO_3_ and BaTiO_3_ or even in mixed perovskites such as Ba_x_Sr_1-x_TiO_3_, due to the fact that the oxygen vacancies do not influence the electron density near Sr atoms. As a result, depending on the individual circumstance, researchers may have options for selecting a suitable perovskite.
(ii) It has been demonstrated that isotropic diffusion is induced by oxygen vacancies in the STO coating layer. Therefore, modulating oxygen concentration during the fabrication process of the coating layer may be an effective strategy to inhibit Zn dendrite growth because homogenous ion flux caused by this isotropic diffusion prevents perpendicular Zn protuberance growth.
(iii) While Zn(II) ion diffusivity in the STO layer increases with an increase in the oxygen vacancy concentration, the vacancies also decrease the structural stability of the STO layer. Therefore, to achieve increased Zn(II) ion diffusivity as well as the structural stability of the STO layer, the oxygen vacancy concentration in the STO layer needs to be optimized.

We believe that a comprehensive understanding of the characteristics of charge imbalances brought on by oxygen vacancies and their effects on Zn(II) ion diffusivity in STO systems is expected to provide insight into the design of new coating layers for anode surfaces with improved Zn(II) ion diffusion properties.

## Figures and Tables

**Table 1:** Maximum and minimum values of differential electron density (Q) caused by oxygen vacancies in the SrTiO_3_ and CaTiO_3_ systems. ΔQ is the difference between the maximum and the minimum values.

System	Maximum Q (e/a0^3^)	Minimum Q (e/a0^3^)	ΔQ (e/a0^3^)
SrTiO_3_	0.035	−0.029	0.064
CaTiO_3_	0.033	−0.031	0.064

## Data Availability

Not applicable.

## Supplementary Materials

The following supporting information can be downloaded at: https://www.mdpi.com/article/10.3390/ma16113957/s1.
