# Supplementary material for: The Effect of Oxygen Vacancies on the Diffusion Characteristics of Zn(II) Ions in the Perovskite SrTiO3 Layer: A Computational Study
Source: Materials (Basel). 2023 May 25;16(11):3957. doi: 10.3390/ma16113957 (PMC10254638; doi:10.3390/ma16113957)
Supplement: Supplementary file 1 [file materials-16-03957-s001.zip › materials-2381822-supplementary.pdf]

Supporting Information

The Effect of Oxygen Vacancies on the Diffusion Characteristics of Zn(II) ions in the Perovskite $SrTiO_3$ Layer: A Computational Study

Yong Nam Ahn

Department of Chemical and Biological Engineering, Gachon University, Seongnam 13120, Gyeonggi, Republic of Korea; yahn@gachon.ac.kr

Interatomic Potential Model

The long-range Coulomb potential, short-range Morse function, and repulsive contribution are the three terms that contributed to the potential employed in the molecular dynamics simulations. Consequently, the formula for the resultant potential is given by

$$U(r) = \frac{z_i z_j e^2}{r} + D_{ij}\left[\left\{1 - e^{-a_{ij}(r-r_0)}\right\}^2 - 1\right] + \frac{C_{ij}}{r^{12}} \qquad (1)$$

Typically, the Coulomb term is removed when simulating bonded interactions in covalent systems using the Morse potential. In this situation, D_{ij}, a_{ij}, and r_0 directly corresponds to the bond dissociation energy, the slope of the potential energy well, and the equilibrium bond distant, respectively. However, D_{ij}, a_{ij}, and r_0 should be considered as parameters in this potential because the Coulomb term is explicitly included.

The partial covalency of oxide systems is handled by a rigid ionic model with partial charges. The oxygen atom's charge has been held constant at -1.2*e*. For the potential to be self-consistent, the partial charges on the cations are compared to the value of the oxygen charge. It should be emphasized once more that these are "effective charges" that must be produce a "effective potential", even though in some circumstances it may be difficult to determine the exact physical meaning of each term. The potential parameters utilized in this study is summarized in Table S1.

Table S1. Potential parameters of Equation (1) for the atomic pairs in $SrTiO_3$ and $CaTiO_3$.

	D_{ij} (eV)	a_{ij} (Å^{-2})	r_0 (Å)	C_{ij} (eV Å^{12})
$Sr^{1.2} – O^{-1.2}$	0.019623	1.886000	3.328330	3.0
$Ca^{1.2} – O^{-1.2}$	0.030211	2.241334	2.923245	5.0
$Ti^{2.4} – O^{-1.2}$	0.024235	2.254703	2.708943	1.0
$O^{-1.2} – O^{-1.2}$	0.042395	1.379316	3.618701	22.0

Differential electron density

To calculate differential electron density brought on by oxygen vacancies, a pristine $SrTiO_3$ (STO) that corresponds to a 2 × 2 × 1 supercell is divided in two systems: (i) a STO system with 16% oxygen vacancies, and (ii) a system that contains the oxygen atoms that were removed from the pristine STO to create the STO system with oxygen vacancies (See Figure S1a). Electron densities for each system are obtained and shown in Figure S1b. Then the differential electron density is calculated by

$$\Delta\rho = \rho_{\mathrm{vac}} - \rho_{\mathrm{prs}} + \rho_{\mathrm{oxy}} \qquad (2)$$

where $\Delta\rho$ is differential electron density and ρ_{vac}, ρ_{prs}, and ρ_{oxy} are the electron densities of the STO with oxygen vacancies, the pristine STO, and the system containing oxygen atoms that were removed from the pristine STO, respectively.

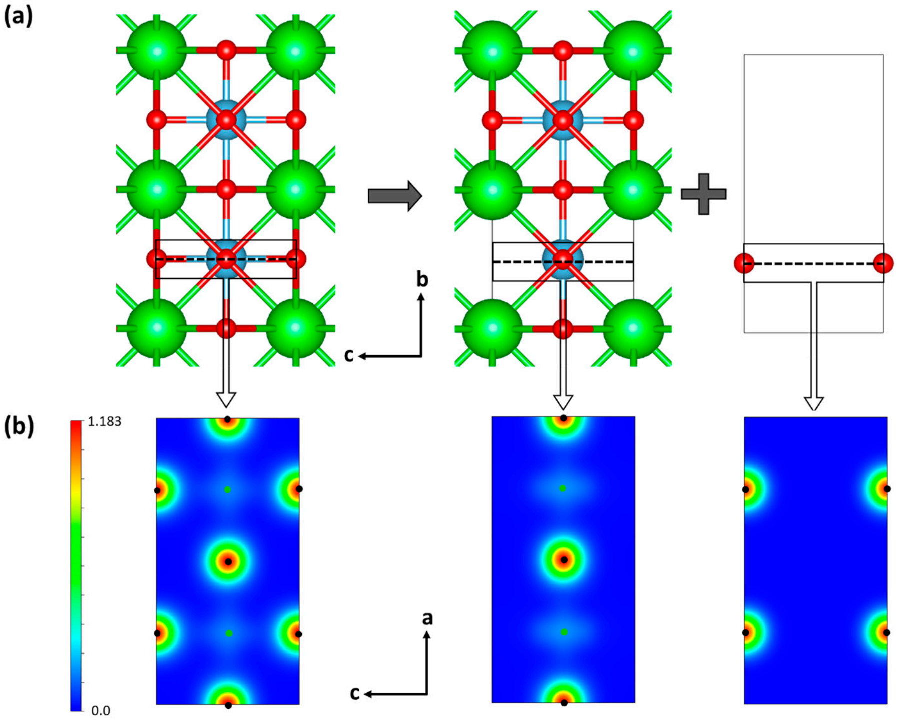

Figure S1. (a) Atomic structures of a pristine $SrTiO_3$, $SrTiO_3$ with 16% oxygen vacancies, and the system containing the oxygen atoms that were removed from the pristine $SrTiO_3$ to

construct the $SrTiO_3$ with oxygen vacancies. Sr, Ti, and O are denoted by green, light blue, and red, respectively. (b) Electron densities of the corresponding atomic structures. On the 2D maps, the locations of Ti and O atoms are denoted by green and black dots, respectively.
